# Progressive deafness–dystonia due to *SERAC1* mutations: A study of 67 cases

**DOI:** 10.1002/ana.25110

**Published:** 2017-12-20

**Authors:** Roeltje R. Maas, Katarzyna Iwanicka‐Pronicka, Sema Kalkan Ucar, Bader Alhaddad, Moeenaldeen AlSayed, Mohammed A. Al‐Owain, Hamad I. Al‐Zaidan, Shanti Balasubramaniam, Ivo Barić, Dalal K. Bubshait, Alberto Burlina, John Christodoulou, Wendy K. Chung, Roberto Colombo, Niklas Darin, Peter Freisinger, Maria Teresa Garcia Silva, Stephanie Grunewald, Tobias B. Haack, Peter M. van Hasselt, Omar Hikmat, Friederike Hörster, Pirjo Isohanni, Khushnooda Ramzan, Reka Kovacs‐Nagy, Zita Krumina, Elena Martin‐Hernandez, Johannes A. Mayr, Patricia McClean, Linda De Meirleir, Karin Naess, Lock H. Ngu, Magdalena Pajdowska, Shamima Rahman, Gillian Riordan, Lisa Riley, Benjamin Roeben, Frank Rutsch, Rene Santer, Manuel Schiff, Martine Seders, Silvia Sequeira, Wolfgang Sperl, Christian Staufner, Matthis Synofzik, Robert W. Taylor, Joanna Trubicka, Konstantinos Tsiakas, Ozlem Unal, Evangeline Wassmer, Yehani Wedatilake, Toni Wolff, Holger Prokisch, Eva Morava, Ewa Pronicka, Ron A. Wevers, Arjan P. de Brouwer, Saskia B. Wortmann

**Affiliations:** ^1^ Translational Metabolic Laboratory, Department of Laboratory Medicine Radboud University Medical Center Nijmegen the Netherlands; ^2^ Department of Audiology and Phoniatrics Children's Memorial Health Institute Warsaw Poland; ^3^ Division of Metabolic Disease, Ege University Medical Faculty, Department of Pediatrics Izmir Turkey; ^4^ Institute of Human Genetics Technische UniversitätMünchen Munich Germany; ^5^ Department of Genetics King Faisal Specialist Hospital and Research Center Riyadh Saudi Arabia; ^6^ Department of Anatomy and Cell Biology College of Medicine, Alfaisal University Riyadh Saudi Arabia; ^7^ Western Sydney Genetics Program, Children's Hospital at Westmead, Sydney New South Wales Australia; ^8^ Discipline of Genetic Medicine & Paediatrics and Child Health, University of Sydney Sydney New South Wales Australia; ^9^ Department of Pediatrics University Hospital Center Zagreb Croatia; ^10^ School of Medicine, University of Zagreb Zagreb Croatia; ^11^ Department of Pediatrics, College of Medicine Imam Abdulrahman Bin Faisal University Dammam Saudi Arabia; ^12^ Division of Inherited Metabolic Diseases, Department of Pediatrics University Hospital of Padua Padua Italy; ^13^ Neurodevelopmental Genomics Research Group, Murdoch Children's Research Institute, and Department of Paediatrics Melbourne Medical School, University of Melbourne Melbourne Victoria Australia; ^14^ Genetic Metabolic Disorders Research Unit and Western Sydney Genetics Program, Children's Hospital at Westmead Sydney New South Wales Australia; ^15^ Discipline of Child and Adolescent Health and Genetic Medicine, Sydney Medical School, University of Sydney Sydney New South Wales Australia; ^16^ Departments of Pediatrics and Medicine Columbia University New York NY; ^17^ Institute of Clinical Biochemistry, Faculty of Medicine, Catholic University of the Sacred Heart Rome Italy; ^18^ Center for the Study of Rare Hereditary Diseases, Niguarda Ca' Granda Metropolitan Hospital Milan Italy; ^19^ Department of Pediatrics Institute of Clinical Sciences, University of Gothenburg, Queen Silvia's Children's Hospital Gothenburg Sweden; ^20^ Childrens Hospital, Klinikum Reutlingen Reutlingen Germany; ^21^ Inborn Errors of Metabolism and Mitochondrial Disease Unit “12 de Octubre” University Hospital, Avenida de Cordoba sn, 28041 Madrid, Spain. Rare Diseases Biomedical Research Centre (CIBERER) Madrid Spain; ^22^ Complutense University Madrid Spain; ^23^ Metabolic Medicine Department Great Ormond Street Hospital for Children National Health Service Foundation Trust, University College London Institute of Child Health London United Kingdom; ^24^ Institute of Medical Genetics and Applied Genomics Tübingen Germany; ^25^ Wilhelmina Children's Hospital Utrecht, University Medical Center Utrecht Utrecht the Netherlands; ^26^ Department of Pediatrics Haukeland University Hospital Bergen Norway; ^27^ Department of Clinical Medicine (K1) University of Bergen Bergen Norway; ^28^ Department of General Pediatrics, Division of Neuropediatrics and Pediatric Metabolic Medicine University Hospital Heidelberg Heidelberg Germany; ^29^ Children's Hospital, University of Helsinki and Helsinki University Hospital Helsinki Finland; ^30^ Research Programs Unit, Molecular Neurology, Biomedicum Helsinki, University of Helsinki Helsinki Finland; ^31^ Department of Biology and Microbiology Riga Stradin's University Riga Latvia; ^32^ Department of Pediatrics Salzburg State Hospitals and Paracelsus Medical University Salzburg Austria; ^33^ Leeds Teaching Hospitals National Health Service Trust Leeds United Kingdom; ^34^ Pediatric Neurology Brussels University Hospital Brussels Belgium; ^35^ Department of Pediatric Neurology Karolinska University Hospital Stockholm Sweden; ^36^ Division of Clinical Genetics, Department of Genetics Kuala Lumpur Hospital Kuala Lumpur Malaysia; ^37^ Department of Clinical Biochemistry, Radioimmunology, and Experimental Medicine Children's Memorial Health Institute Warsaw Poland; ^38^ University College London Great Ormond Street Institute of Child Health London United Kingdom; ^39^ Department of Pediatric Neurology Red Cross War Memorial Children's Hospital Cape Town South Africa; ^40^ Department of Neurodegeneration Hertie Institute for Clinical Brain Research, University of Tübingen Tübingen Germany; ^41^ German Center for Neurodegenerative Diseases (DZNE) Tübingen Germany; ^42^ Department of General Pediatrics Münster University Children's Hospital Münster Germany; ^43^ Department of Pediatrics University Medical Center Eppendorf Hamburg Germany; ^44^ Reference Center for Inherited Metabolic Diseases, AP‐HP, Robert Debré Hospital, University Paris Diderot‐Sorbonne Paris Cité, Paris, France AND INSERM U1141 Paris France; ^45^ Department of Human Genetics Radboud University Medical Center Nijmegen the Netherlands; ^46^ Metabolic Unit Dona Estefânia Hospital Lisbon Portugal; ^47^ Wellcome Centre for Mitochondrial Research Institute of Neuroscience, The Medical School, Newcastle University Newcastle upon Tyne United Kingdom; ^48^ Department of Medical Genetics Children's Memorial Health Institute Warsaw Poland; ^49^ Division of Metabolic Diseases Hacettepe University Children's Hospital Ankara Turkey; ^50^ Birmingham Children's Hospital Birmingham United Kingdom; ^51^ Nottingham University Hospitals National Health Service Trust, Nottingham Children's Hospital Nottingham United Kingdom; ^52^ Institute of Human Genetics, Helmholtz Center Munich Neuherberg Germany; ^53^ Hayward Genetics Center and Department of Pediatrics Tulane University Medical School New Orleans LA; ^54^ Department of Pediatrics, Nutrition and Metabolic Diseases Children's Memorial Health Institute Warsaw Poland; ^55^ Donders Institute for Brain, Cognition, and Behavior, Radboud University Medical Center Nijmegen the Netherlands

## Abstract

**Objective:**

3‐Methylglutaconic aciduria, dystonia–deafness, hepatopathy, encephalopathy, Leigh‐like syndrome (MEGDHEL) syndrome is caused by biallelic variants in *SERAC1*.

**Methods:**

This multicenter study addressed the course of disease for each organ system. Metabolic, neuroradiological, and genetic findings are reported.

**Results:**

Sixty‐seven individuals (39 previously unreported) from 59 families were included (age range = 5 days–33.4 years, median age = 9 years). A total of 41 different *SERAC1* variants were identified, including 20 that have not been reported before. With the exception of 2 families with a milder phenotype, all affected individuals showed a strikingly homogeneous phenotype and time course. Severe, reversible neonatal liver dysfunction and hypoglycemia were seen in >40% of all cases. Starting at a median age of 6 months, muscular hypotonia (91%) was seen, followed by progressive spasticity (82%, median onset = 15 months) and dystonia (82%, 18 months). The majority of affected individuals never learned to walk (68%). Seventy‐nine percent suffered hearing loss, 58% never learned to speak, and nearly all had significant intellectual disability (88%). Magnetic resonance imaging features were accordingly homogenous, with bilateral basal ganglia involvement (98%); the characteristic “putaminal eye” was seen in 53%. The urinary marker 3‐methylglutaconic aciduria was present in virtually all patients (98%). Supportive treatment focused on spasticity and drooling, and was effective in the individuals treated; hearing aids or cochlear implants did not improve communication skills.

**Interpretation:**

MEGDHEL syndrome is a progressive deafness–dystonia syndrome with frequent and reversible neonatal liver involvement and a strikingly homogenous course of disease. Ann Neurol 2017;82:1004–1015

The first clinical description of 4 individuals with MEGDEL (3‐methylglutaconic aciduria, dystonia–deafness, encephalopathy, Leigh‐like) syndrome was published in 2006.^1^ In 2012, biallelic variants in *SERAC1* (serine active site containing 1) were shown to cause this autosomal‐recessive deafness–dystonia disorder.^2^ Soon afterward, with the description of liver involvement as an additional clinical feature, hepatopathy was incorporated into the acronym (MEGDHEL; Mendelian Inheritance in Man [MIM] #614739).^3^

*SERAC1* encodes a protein with a serine–lipase domain, which is a member of the PGAP‐like protein domain family. SERAC1 is localized at the mitochondria‐associated membranes, the contact sites between endoplasmic reticulum and the mitochondrial interface, which are crucial for phospholipid exchange.^4^ The enzyme is involved in the remodeling of the phospholipid phosphatidylglycerol, the precursor of both cardiolipin, essential for proper mitochondrial function, and bis(monoacylglycerol)phosphate, essential for intracellular cholesterol trafficking, respectively.^2^

SERAC1 deficiency is one of the signature disorders of an emerging and rapidly growing new class of disorders affecting the biosynthesis and remodeling of complex lipids.^5^, ^6^ So far, 2 further genetic syndromes have been linked to the cardiolipin biosynthetic pathway: AGK deficiency (Sengers syndrome, consisting of 3‐methylglutaconic aciduria [3‐MGA‐uria], cardiomyopathy, and cataracts, MIM #212350) and TAZ deficiency (Barth syndrome, consisting of 3‐MGA‐uria, cardiomyopathy, and neutropenia, MIM #302060).

Here, we describe the results of a detailed systematic, multicenter study of the genetic, biochemical, radiological, and clinical findings of a cohort of 67 individuals (including 39 previously unreported cases) with MEGDHEL syndrome. We present 20 novel variants in *SERAC1* and a complete phenotypic description, thereby facilitating diagnosis, and providing a proper prognosis for patients with this disorder.

## Patients and Methods

### Informed Consent

All procedures followed were in accordance with the ethical standards of the Helsinki Declaration of 1975 as revised in 2000.^7^

### Cohort and Phenotypic Evaluation

All individuals in this study had rare biallelic variants in *SERAC1* and typical phenotypic findings leading to the diagnosis of MEGDHEL syndrome (detailed in the Results section). Their respective physicians completed a questionnaire concerning the course of disease for each organ system, together with metabolic, radiological, and genetic findings. GraphPad (La Jolla, CA) Prism7 was used for Kaplan–Meier survival analysis.

### Biochemical Investigations in Tissues and Specimens of Affected Individuals

Urinary organic acid analysis; serum/plasma amino acid analysis; histological and immunohistochemical evaluation, measurement of the oxidative phosphorylation system (OXPHOS), and quantitation of mtDNA in muscle, liver, or cultured fibroblasts; and filipin staining in cultured fibroblasts were performed using standard methods as described before.^2^, ^8^

### Identification of *SERAC1* Variants

Variants were found either by Sanger sequencing, exome sequencing as previously described,^2^, ^9^, ^10^, ^11^, ^12^, ^13^, ^14^, ^15^ or genome sequencing.^16^ All variants found in individuals, and carrier status of parents, were confirmed by Sanger sequencing (details available upon request). The deletion of exon 4 to exon 8 in P18 was identified by genomic quantitative polymerase chain reaction (qPCR) as described previously.^17^ qPCR primers of exon 3 through exon 13 of *SERAC1* are available upon request.

## Results

### Genetic Findings and Incidence of MEGDHEL Syndrome

**Figure 1:**
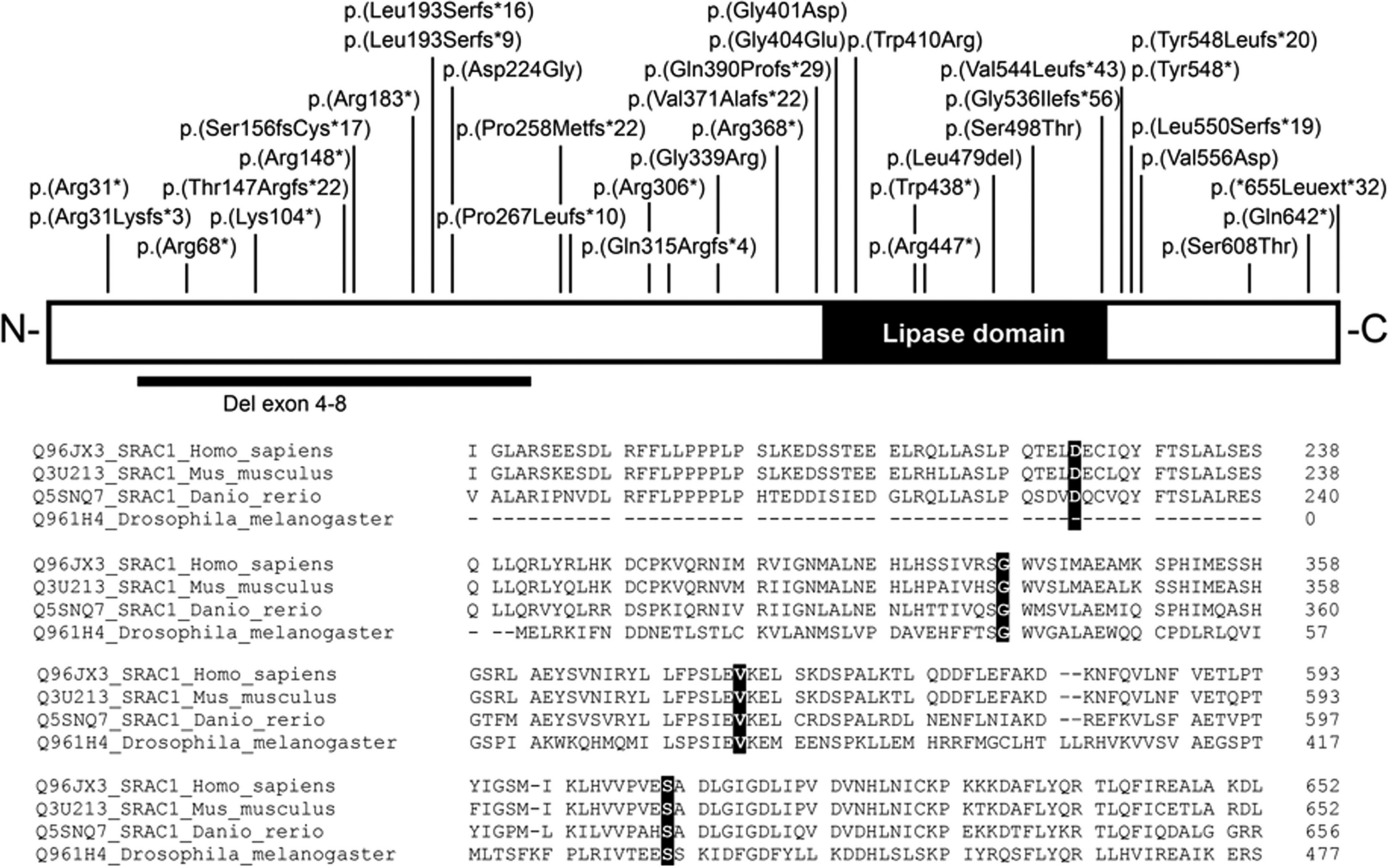
The upper panel shows a schematic representation of the human SERAC1 protein with the positions of all variants identified. The black box represents the lipase/esterase domain. The lower panel shows the Cross‐species alignment. Clustal Omega^34^ targeted the protein sequences directly surrounding the 4 missense variants, p.(Asp224Gly), p.(Gly339Arg), p.(Val556Asp), and p.(Ser608Thr). The changes are highlighted by the black boxes; all conserved down to the zebrafish. Protein accession numbers used for alignment are given before the sequences and include the specific species. The position of the last amino acid residue in each row is given right after the respective sequences.

A total of 41 different *SERAC1* (NM_032861.3) variants were identified in the 67 individuals described, including 20 that have not been reported before (Fig 1, Supplementary Table 1). Fifteen individuals had compound heterozygous variants and 52 a homozygous variant. Variants were categorized as frameshift (n = 13), nonsense (n = 11), missense (n = 8), canonical splice site (n = 3), splice site (n = 3), frameshift/canonical splice site (n = 1), extension (n = 1), and in‐frame deletion variants (n = 1). These variants were predicted to result either in nonsense‐mediated mRNA decay (n = 29), a truncated protein (n = 2), impaired lipase function (n = 5), or extension of SERAC1 of 32 amino‐acid residues (n = 1). For 4 missense variants, the effect on protein level is uncertain, as they are not located in the lipase domain (see Fig 1). However, they affected amino acids that are conserved down to zebrafish, indicating that they are essential for proper protein function.

Four variants were found multiple times (see Fig 1). The c.1822_1828 + 10delinsACCAACAGG, p.(?) was detected in 14 European families, indicating that it could be a founder variant. Likewise, the c.1493G>C; p.(Ser498Thr) was found in 4 other European families; for 2 of them, common ancestry has been shown previously via haplogroup analysis.^2^ The c.1403 + 1G>C; p.(Arg446*) was detected in 5 families (and 1 additional from the literature)^18^ The c.202C>T; p.(Arg68*) was documented in 3 families from the greater Mediterranean area, and c.442C>T; p.(Arg148*) was seen in 6 families from Turkey, Saudi Arabia, and China, which may reflect distribution of the variant along the Silk Route.

Based on the prevalence of deleterious *SERAC1* alleles in the normal population (ExAC database; Lek et al^19^, we estimate that approximately 27 children with MEGDHEL will be born each year worldwide (Supplementary Table 2).

### General Characteristics of Affected Individuals and Survival

A total of 67 individuals from 59 families were included, of whom 28 individuals have been published previously (P1–15,^2^ P35,^10^ P42,^11^ P49,^20^ P50,^12^ P51–52,^13^ P58,^14^ P60–64,^21^ and P66.^15^

Most of the individuals reported here are of European ancestry (see Supplementary Table 1, n = 41), although we have also ascertained individuals from Africa (n = 4), Asia (n = 12), the Middle East (n = 12), and Australia (n = 1), showing that MEGDHEL syndrome is a panethnic condition.

In 38 of 59 (64%) families, consanguinity was reported. The male to female ratio was 1:1.3 and the median age of individuals (as of September 2016) in our cohort was 9 years (range = 5 days–33.4 years).

The median age at diagnosis (the age at the date of the genetic report was used for calculations; if individuals were deceased, the age at death was used for calculation) was 7.2 years. Thirty‐two individuals were diagnosed with targeted *SERAC1* Sanger sequencing (median age at diagnosis = 5.7 years), 26 via next generation sequencing techniques (7.2 years), and 9 via family screening by Sanger sequencing (3.7 years). Seven individuals were diagnosed at <1.5 years, of whom 2 had liver failure as the dominating finding, 2 had the typical neurological signs and symptoms, 1 was based on the magnetic resonance imaging (MRI), and 2 were diagnosed due to various reasons and a positive family history.

**Figure 2:**
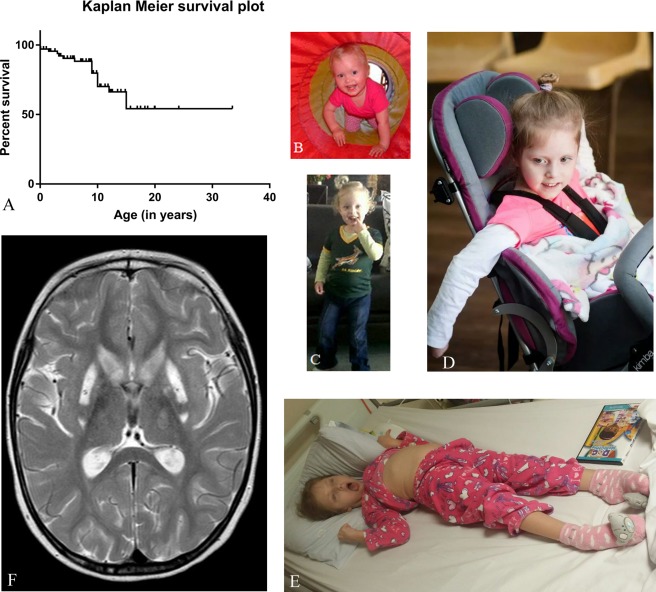
(A) Kaplan–Maier Survival plot showing overall survival for 67 individuals. (A–E) clinical photos of P28 at the age (B) 18 months, (C) 3 years, (D) 5 years, and (E) 8 years. Note the progressive spasticity and the dystonic posture of the limbs. The patients’ parents gave written permission for showing the face of their child. (F) T2‐weighted magnetic resonance image of the same individual at the age of 5 years showing bilateral ganglia involvement sparing the central putamen (“putaminal eye”).

Sixteen individuals passed away at a median age of 9 years (range = 5 days–16 years; for Kaplan–Meier survival curves, see Fig 2). The main causes of death were respiratory infections (10/16 = 63%) and multiorgan failure (1 child), and were unknown in 5 cases.

### Major Clinical Features

#### Pregnancy and Delivery

Problems during pregnancy (data were available for 55 pregnancies) were uncommon; 8 pregnancies (8/55, 15%) were reported as having been complicated by intrauterine growth retardation, 1 with oligohydramnios and none with polyhydramnios; decreased movements were described in 1 fetus. Forty‐six individuals were delivered by a normal vaginal delivery (84%) and 9 by Caesarean section (16%; no details about the indication were available).

#### Neonatal Course

Forty‐seven individuals (47/67 = 70%) presented in the neonatal period and required inpatient observation or treatment. Twenty‐two of 45 (49%) had suspected neonatal sepsis, and 30/62 (48%) had severe liver dysfunction. Twenty‐nine individuals had recurrent hypoglycemia (glucose values < 2mmol/l in 29/65 = 44%), 9/53 (17%) were ventilated due to respiratory insufficiency, and 31/67 (46%) were reported to have other neonatal adaptation problems. Two individuals died in the neonatal period, 1 due to respiratory insufficiency at 5 days of age (P7) and 1 due to multiorgan failure at 6 days of age (P31).

### Neurological Features, Developmental Milestones, Intellectual Abilities, and Daily Living

Neurological features followed a specific pattern in almost all individuals. The first signs were delayed motor development (57/66 = 86%) and muscular hypotonia (59/65 = 90%), recognized at a median age of 6 months. Twenty‐eight of 59 individuals (47%) presented with axial hypotonia, 17 with generalized hypotonia (17/59 = 29%); for 14, no details were available. Previously obtained skills were lost, starting from a median of 12 months of age, in the majority of individuals (50/67 = 75%). Progressive spasticity of the limbs developed at a median age of 15 months (53/65 = 82%, range of onset = 1–48 months), and dystonia at a median age of 18 months (53/65 = 82%, range of onset = 1–84 months). Dystonia mainly involved the upper extremities and to a lesser extent the lower extremities. Typically, individuals presented oropharyngeal dyskinesia with repetitive protrusion of the tongue, dysphagia, and excessive drooling (34/59 [58%]). For clinical photographs, see Figure 2.

Seventy‐eight percent (26/38) of the individuals never learned to walk. Of the 12 individuals who walked independently, 3 lost this skill within 1 year, and 2 had preserved ambulation up to the age of 6 and 7 years, respectively; 5 individuals from 1 family (Family 56, P60–64) and another female (P56) have preserved ambulation into their 20s and up to 8 years, respectively. With disease progression, 39% (19/49) of individuals developed scoliosis. Epilepsy was reported in 23/66 individuals (35%). All 67 individuals had intellectual disability, varying in severity (severe, 37/51 = 73%; moderate, 8/51 = 16%; mild, 6/51 = 12%; data on severity were lacking for 16 individuals). Nearly all individuals (62/67 = 93%) were completely dependent on others for all activities of daily living.

It is important to note that 1 family of 5 affected individuals (Family 56, P60–64, details will be reported separately^21^ and 1 female individual (P67) had a milder course of disease. In Family 56, the presentation was spasticity and loss of skills starting at the age of 5 years in 2 siblings, whereas spasticity did not start before adolescence in 2 other siblings and is still absent in the fifth sibling at the current age of 11 years. Only 2 of the siblings showed dystonia. They all learned to walk; the oldest lost this ability at the age of 12 years, and the other individuals are still ambulatory at ages between 12 and 24 years. They all have a mild intellectual disability, are able to communicate with words, with the exception of the oldest individual, who has lost this ability. They never displayed hepatic dysfunction and have normal hearing.

The female (P67) presented with delayed development, general muscular hypotonia, and failure to thrive at the age of 3 years. She is currently aged 8 years, is able to walk independently, has a mild intellectual disability, but is in a mainstream school with additional support. She never had any significant hepatic dysfunction, has normal hearing, and does not exhibit spasticity or a movement disorder.

### Neuroradiological Findings

Individuals with MEGDHEL syndrome show a characteristic MRI pattern with 5 distinctive disease stages affecting the basal ganglia and especially the putamen; these data have been reported in detail separately.^22^ First, in stage 1, T2 signal changes of the pallidum are seen. Stage 2 is characterized by swelling of the putamen and caudate nucleus. The dorsal putamen contains an “eye” that showed no signal alteration and so seemed to be spared during this stage of the disease. From stage 3 onward, the “putaminal eye” (see Fig 2) gradually decreases, mirroring progressive basal ganglia dysfunction. Finally, stage 4 is characterized by shrunken basal ganglia, which further atrophy in stage 5. Brain MRI studies were available for 55/67 individuals. In all individuals, alterations of the basal ganglia at different stages were observed. In 29 of 55 individuals, the pathognomic putaminal “eye” was reported, which is visible in stages 2 and 3.

### Hearing Impairment and Speech Development

Forty of 52 individuals passed the neonatal hearing screen. Forty‐eight of 61 individuals (79%) were diagnosed with sensorineural hearing impairment; this was diagnosed in the neonatal period in 11/48 (23%), before the age of 1 year in 7/48 (15%), and later in 25/48 (52%). In 56% of individuals (34/61), speech was completely absent. Four children (7%) could use sounds to communicate (dis)comfort; an additional 5 had lost this skill (8%). Eleven of the 61 individuals (18%) were able to use words for a limited period of time (maximum = 1.5 years) before losing this skill. Seven individuals (11%) were still able to communicate with words at the ages of 8 years (P48, P67) and 12 to 24 years (Family 56, P60–64), respectively.

Thirty‐two children were fitted with hearing aids. Appropriate tolerance of reinforcement/sounds was seen in all cases, with improvement of formal hearing test results. The behavioral reactions during fitting included smiling, articulation of sounds, or quieting. However, 10 individuals did not continue to tolerate the hearing aids and showed agitated and frightened behavior. Another 4 individuals underwent cochlear implantation, which was also not tolerated. None of these 36 individuals showed improvement in speech development following auditory augmentation, but the parents of 4 children reported improved interaction with the environment. The mechanism of hearing impairment in MEGDHEL is probably sensorineural (cochlear), but the coexistence of a neural or central component is likely. The limited data on the age of hearing loss appearance and progression mean that it is currently impossible to draw firm conclusions about the pathomechanism. A simple explanation that this reflects high energy demand of sensory tissues is inadequate, as hearing loss occurs selectively only in some mitochondrial disorders and is absent in others with comparable course and severity.

An even more complex clinical finding is the near complete lack of speech development in most MEGDHEL cases. Although the hearing loss seems to be the major cause of loss of verbal abilities, a multifactorial contribution of intellectual impairment, oropharyngeal dyskinesia, extrapyramidal movements of the tongue, dysphagia, and drooling is almost certain. Furthermore, the characteristic basal ganglia involvement could be important. In the procedural/declarative model of language learning, an important role for the basal ganglia in the assembly of phonemes into words is suggested.^23^

### Visual Impairment

A total of 26 individuals of 62 (42%) were reported to have impaired vision. Four of 64 (6%) individuals had retinal pigmentary changes. Fourteen of 55 individuals (25%) had signs of optic atrophy documented on fundoscopy or MRI, which is the common morphological endpoint of any disease‐causing axonal degeneration. The underlying pathophysiology is speculative but in line with the neuronal degeneration of basal ganglia. A possible explanation is mitochondrial dysfunction in the broadest sense impairing axonal transport and leading to axonal degeneration.^24^

### Liver Involvement

Severe neonatal liver dysfunction was reported in 30/62 (48%) individuals. Nine of these 30 (30%) individuals fulfilled the criteria of neonatal liver failure (elevated aspartate aminotransferase (ASAT), alanine aminotransferase (ALAT), and/or conjugated bilirubin, disturbed coagulation with international normalized ratio > 2, and encephalopathy). Seven individuals did not meet the full definition of liver failure but presented hyperammonemia (maximum level = 600 µmol/l, reference range < 100 µmol/l), and were treated with protein restriction and ammonia scavengers, and 1 individual additionally underwent hemofiltration. Two individuals were given galactose‐free formulas in the neonatal period due to the combination of jaundice, neurological features, and a suspicion of sepsis, raising a possible diagnosis of galactosemia.

Beyond the neonatal period, no individual exhibited features of liver failure; however, signs of hepatic dysfunction were frequently reported. During the first year of life, 24/50 (48%) individuals were reported with transient cholestasis and jaundice. Hepatomegaly at any age during their lifetime was reported in 17/64 (27%) individuals, and disturbed coagulation tests were documented in 18 of 55 individuals (33%), but only 1 (1/55) had subsequent bleeding problems.

During the course of a life ASAT/ALAT were transiently increased on multiple occasions in 42/55 (76%) individuals (range = 1.5–60‐fold elevated). The duration of elevation lasted for up to 9 months and was most prominent during the first year of life.

A liver biopsy was obtained from 11 individuals and showed nonspecific histopathological changes (fibrosis n = 8, cholestasis n = 5, steatosis n = 6, and ductopenia n = 1) in 8/11 cases (73%). The OXPHOS system was evaluated in 6 liver biopsies, and 3 of these samples showed deficiencies of different OXPHOS enzymes. Mild mtDNA depletion was measured in 3 of 4 liver biopsies compared to age‐matched controls.

### Renal, Cardiac, and Other Organ Involvement

Eight individuals showed impaired tubular function (8/66 = 12%; eg, P35^10^) mostly transient and in the neonatal period. One individual had hypophosphatemic rickets, possibly due to incapacity of the renal tubules to reabsorb phosphate (further evaluation was not performed).

In 5 of 67 individuals (7%), cardiac abnormalities were reported. These were congenital heart defects (mild pulmonary stenosis, patent foramen ovale, and a small atrium septum defect were reported in 1 individual each) without hemodynamic consequences. In 2 individuals suffering multiorgan failure during an infection, left ventricular hypertrophy with good function (n = 1) and paroxysmal bradycardia (n = 1) were seen.

Many individuals suffered recurrent respiratory infections (28/66 = 42%), which were also the main cause of death. No immunological problems have been reported in our cohort. We consider it likely that the combination of scoliosis, gastroesophageal reflux, insufficient clearing of the airway with pharyngeal pooling of secretions, and subsequent microaspirations causes the frequent respiratory infections.

Failure to thrive and feeding problems were a common and concomitant problem (52/66 individuals = 79%). Thirty‐seven of 58 individuals (64%) were given tube feeding.

### Disease Management

More detailed data on drug treatment were available for 22 individuals. Oral baclofen was given to 15 individuals. It was reported as having a positive effect in 11 individuals, whereas in 2 no change was observed, and in another 2, clinical deterioration of spasticity was seen. Four individuals received oral L‐dopa, with improvement of the movement disorder in 1 case, no clinical improvement in 2 individuals, and worsening of dystonia in the fourth case.

Many individuals were reported to be on proton pump inhibitors (indication was gastroesophageal reflux) or macrogols (constipation), although detailed data were not available. Cessation of seizures was reported following antiepileptic drugs in 10 individuals; the other individuals with epilepsy were given only rescue medicines such as midazolam, which was well tolerated. Four individuals were reported to take melatonin for sleeping problems with good effect.

“Multivitamin cocktails” containing for example coenzyme Q_10_, riboflavin, and biotin, either prescribed by their physician or as over‐the‐counter drugs in different combinations and with different doses, were taken by many individuals. Reliable data were available for only 5 individuals, for whom none of the caretakers or physicians reported any discernible clinical difference.

Treatment of drooling in MEGDHEL syndrome has been evaluated separately.^25^ In addition to the described successful surgical correction, P28 and P65 were given atropine 0.5% eye drops orally (1 drop every 6 hours), which reduced drooling to a normal level without any side effects.

### Metabolic Findings in Blood and Urine

The most striking metabolic finding in MEGDHEL syndrome was the increased urinary excretion of 3‐MGA. Sixty‐one of 62 (98%) individuals had 3‐MGA‐uria (for 47 individuals, quantitative values of several investigations were available; median lowest values = 63mmol/mol creatinine, median highest values = 141mmol/mol creatinine, reference range < 20mmol/mol creatinine). Remarkably, in 1 individual no 3‐MGA‐uria was detected (P41). In all patients for whom multiple 3‐MGA measurements were available, these fluctuated up to a 3‐fold increase. Serum lactate levels were increased in 51 of 61 (84%) individuals; the median of the respective maximum values was 5mmol/l (range = 1–53.3, normal value < 2mmol/l).

### Evaluation of Tissues (Muscle, Liver, Cultured Fibroblasts)

Histological and immunohistochemical evaluation revealed nonspecific alterations (eg, fiber size disproportion, mild accumulation of lipids or glycogen) in 24/37 (65%) available muscle biopsies. Eleven of 20 (55%) muscle samples showed abnormalities by electron microscopy; these were also nonspecific changes, including abnormally shaped or damaged mitochondria, tubular aggregations^11^ in the subsarcolemmal regions, fatty vacuoles, and oil droplets. Filipin staining of fibroblasts was abnormal in 6 of 10 (60%) individuals.

In 14/32 (44%) muscle biopsies, a deficiency of 1 or more of OXPHOS enzymes was observed. OXPHOS complex deficiencies were also found in 8/22 (36%) individuals’ fibroblast cell lines and in 3 of 6 liver biopsies. No specific pattern of deficiencies was observed. Additionally, mild mtDNA depletion compared to age‐matched controls was reported in 3 of 4 liver biopsies. As the underlying protein defect is not thought to influence mtDNA translation, this might well be a secondary effect.

## Discussion

We describe the results of a systematic, multicenter study on the genetic, clinical, neuroradiological, metabolic, and biochemical findings of a cohort of 67 individuals with MEGDHEL syndrome. Additionally, 7 individuals—who were not included in this study—have been reported in the literature with a comparable course of disease (Table 1, Supplementary Table 3).^3^, ^18^, ^26^

**Table 1:** Most Frequent Clinical, Radiological, and Metabolic Findings in Individuals with MEGDHEL Syndrome

Finding	Cohort, n = 67	Literature, n = 7	Total, n = 74	Median Age of Onset, yr^a^
Ethnicity	b	c		
Any neonatal problem	47/67 (70%)	7/7 (100%)	54/74 (73%)	
Neonatal hypoglycemia	29/65 (44%)	5/5 (100%)	34/70 (49%)	
Severe neonatal liver dysfunction	30/62 (48%)	5/5 (100%)	35/67 (52%)	
Neonatal liver failure	15/65 (23%)	7/7 (100%)	22/72 (30%)	
Muscular hypotonia	59/65 (91%)	5/5 (100%)	64/70 (91%)	6 mo
Loss of skills	50/67 (75%)	n/a	50/67 (75%)	12 mo
Progressive spasticity	53/65 (82%)	4/5 (80%)	57/70 (81%)	15 mo
Dystonia	53/65 (82%)	4/5 (80%)	57/70 (81%)	18 mo
Oropharyngeal dyskinesia, protrusion of the tongue	34/59 (58%)	n/a	34/59 (58%)	
Never learning to walk	26/38 (68%)	n/a	26/38 (68%)	
Sensorineural hearing loss	48/61 (79%)	5/5 (100%)	53/66 (80%)	
Never learning to speak	34/59 (58%)	n/a	34/59 (58%)	
Moderate to severe intellectual disability	45/51 (88%)	1/1 (100%)	46/52 (88%)	
Epilepsy	23/66 (35%)	4/6 (67%)	27/72 (38%)	
MRI: basal ganglia involvement	55/56 (98%)	5/5 (100%)	60/61 (98%)	
Optic atrophy	14/55 (25%)	0/1 (0%)	14/56 (25%)	
3‐methylglutaconic aciduria	61/62 (98%)	6/6 (100%)	67/68 (99%)	
Lactic acidosis	51/61 (84%)	6/6 (100%)	57/67 (85%)	
Positive filipin staining in fibroblasts	6/10 (60%)	2/2 (100%)	8/12 (67%)	

a In our cohort.

b Europe (n = 41): Turkey (n = 17); Poland (n = 6); Sweden (n = 4); Finland, Spain (n = 2); Latvia, Ukraine, Rumania, Italy, Croatia, Portugal, the Netherlands, Belgium, Germany, (¼ German, ¼ Curacao, ½ Polish; n = 1). Africa (n = 4): Somalia (n = 2), South Africa, French African country (no details available; n = 1). Asia (n = 9): Pakistan (n = 3); India (n = 2); Malaysia (n = 2); Afghanistan, Bangladesh, China (n = 1). Middle East (n = 12): Saudi Arabia (n = 7); Iraq (n = 5). Australia (n = 1).

c Arab Muslim (n = 2), Druze (n = 2), Palestine (n = 1), Pakistan (n = 2).

MEGDEL = 3‐methylglutaconic aciduria, dystonia–deafness, encephalopathy, Leigh‐like syndrome; MRI = magnetic resonance imaging; n/a = not available.

We report 20 new sequence variants, which, together with the already known sequence variants, make a total of 44 different known *SERAC1* sequence variants. The sequence variants are located throughout the whole gene, with no hotspots. There are more loss‐of‐function (stop, frameshift, and splice site) variants than missense variants, with a respective ratio of 30:8, suggesting that most missense variants are nondeleterious. This could consequently suggest that milder clinical phenotypes are currently underdiagnosed. The missense sequence variants that we have identified inside and outside the highly conserved lipase domain are all conserved down to the zebrafish, indicating that they are essential for SERAC1 function, thus supporting causality.

We here report 1 family^21^ and—for the first time—1 female and with a significantly milder course of disease than known before. The mild phenotype in the family might be related to the nature of the respective *SERAC1* sequence variant, a noncanonical splice site change, c.91 + 6T>C, for which aberrant splicing was proven.^21^ The reason for the mild course in the female cannot be explained on genetic grounds.

MEGDHEL syndrome is best described as a progressive deafness–dystonia syndrome with frequent liver involvement. Despite the severity of neurological impairment, most affected individuals survive into adulthood. The clinical phenotype of the 67 individuals reported here and the 7 reported in the literature is strikingly homogenous with regard to the specific clinical findings reported (eg, hearing impairment, basal ganglia involvement), the age of onset of these, and their severity. Besides the neuro(radiological) findings including affection of the sense organs (hearing loss, optic atrophy) and the liver involvement that is of clinical impact only in the neonatal period, no other organ systems are involved in MEGDHEL syndrome. We can only speculate about the cause for this distribution of organ involvement. With regard to the homogeneity of the clinical phenotype, we cannot exclude that our data may be biased, as the vast majority of patients were only diagnosed after occurrence of neurological symptoms. However, the individuals in our cohort diagnosed in the first months of life as well as the ones with the milder phenotypes also developed the characteristic combination of signs and symptoms. Furthermore, from our experience with exome sequencing for rare pediatric disorders (the Munich databases [12,000 exomes] encompass, eg, >1,000 exomes of suspected mitochondrial cases, >1,000 intellectual disability cases, >500 epilepsy cases, and >100 acute liver failure cases), we did not identify individuals with different or partial phenotypes.

We classify MEGDHEL syndrome primarily as a disorder of the biosynthesis of complex lipids with secondary mitochondrial dysfunction, although MEGDHEL syndrome displays typical findings and the progressive course of a mitochondrial disorder, including lactic acidosis and 3‐MGA‐uria.^27^ However, when measuring the OXPHOS system in tissues of affected individuals, the mitochondrial dysfunction greatly varies, and often measurements are unremarkable. The same holds for the disturbed cholesterol trafficking seen in MEGDHEL syndrome (visualized by abnormal filipin staining of fibroblasts of affected individuals), which may underlie the neonatal liver involvement. This combination of early liver pathology followed by later onset neurological sequelae is also seen in Niemann–Pick disease, type C. Neonatal liver failure is also a frequent finding of mtDNA depletion disorders (eg, due to variants in *POLG, DGUOK, MPV17*) and in some of the liver biopsies of affected MEGDHEL individuals mild mitochondrial depletion was reported. Based on the pathophysiology, we would not consider this a primary effect of *SERAC1* variants, but would regard this as a secondary effect, as it has been reported similarly in several nonmitochondrial disorders (eg, propionic academia).^28^

The differential diagnosis of MEGDHEL syndrome depends on the “key” sign or symptom an individual presents; Table 2 provides an overview. In general, MEGDHEL should be considered in individuals with (1) neonatal adaptation problems in combination with hypoglycemia and/or (reversible) liver failure, (2) the rare clinical combination of deafness and dystonia, (3) a clinical course with rapid regression and development of spasticity and dystonia starting around 18 months of age, and (4) the pathognomonic finding of the “putaminal eye” on cerebral MRI. In all these cases, urinary organic acid analysis and appropriate genetic testing for *SERAC1* variants should be performed.

**Table 2:** The Differential Diagnoses of MEGDEL Syndrome Based on Key Features

Key Features	Diagnoses
(Reversible, neonatal) liver failure, lactic acidosis	Mitochondrial DNA depletion syndromes (*MPV17* [MIM #256810], *DGUOK* [MIM #251880], *TFAM* [MIM #617156], *TWNK* [MIM #271245], *POLG* [MIM #203700], AR)
	Transient infantile liver failure due to variants in *TRMU* (MIM #613070, AR; caveat: often presents only in 2nd/3rd months of life)
	Niemann–Pick disease type C (*NPC1* (MIM #257220, AR; caveat: no lactic acidosis)
Isolated, significantly (>40mmol/mol creatinine, reference < 20) and repetitively elevated urinary 3‐methylglutaconic acid without elevation of 3‐hydroxyisovaleric acid	TAZ deficiency (Barth syndrome, *TAZ*, MIM #302060, XLR): (cardio)myopathy, neutropenia, growth failure, DD
	OPA3 deficiency (Costeff syndrome, *OPA3*, MIM #258501, AR): optic atrophy, extrapyramidal symptoms (ataxia), DD
	DNAJC19 deficiency (DCMA syndrome, *DNAJC19*, MIM #610198, AR): dilated cardiomyopathy, ataxia, growth failure, endocrinological features, ID/DD^27^
	TMEM70 deficiency (*TMEM70*, MIM #614052, AR): neonatal hyperammonemia, (cardio)myopathy, metabolic crises, ID/DD
	CLPB deficiency (*CLPB*, MIM #616271, AR): cataracts, neutropenia, variable neurological course^28^
	AGK deficiency (Sengers syndrome, *AGK*, MIM #212350, AR): cardiomyopathy, cataracts; isolated cataracts
	HTRA2 deficiency (*HTRA2*, AR): neonatal encephalopathy, neutropenia, muscular hypo‐ and hypertonia, seizures, ID/DD^29^, ^30^
	TIMM50 deficiency (*TIMM50*, AR): intractable seizures, ID/DD^31^
Deafness–dystonia	SUCLA2 deficiency (*SUCLA2*, MIM #612073, AR), marker: mildly increased methylmalonate combined with elevated carnitine esters (C4DC) in both plasma and urine
	Mohr–Tranebjærg syndrome (*TIMM8A*, MIM #241080, XLR)
	Woodhouse–Sakati syndrome (*DCAF17*, MIM #241080, AR)
	Deafness, dystonia and cerebral hypomyelination (*BCAP31*, MIM #300398, XLR)

AR = autosomal recessive; DCMA = dilated cardiomyopathy with ataxia; DD = developmental delay; ID = intellectual disability; MEGDEL = 3‐methylglutaconic aciduria, dystonia–deafness, encephalopathy, Leigh‐like syndrome; MIM = Mendelian Inheritance in Man; XLR = X‐linked recessive.

MEGDHEL syndrome is one of the signature disorders of a new group of disorders with a defect in the biosynthesis of phospholipid; Table 3 provides an overview of the subgroup of these disorders with central nervous system involvement.^29^, ^30^, ^31^, ^32^, ^33^

**Table 3:** Overview of Disorders of the Biosynthesis and Remodeling of Phospholipids with Central Nervous System Involvement

Predominant Clinical Finding	Disorders (gene in which mutations are found)
Hereditary spastic paraparesis	Calcium‐independent phospholipase A2γ (*PLA2G6*), fatty acid elongase ELOVL4 (*ELOVL4*), cytochrome P450 hydroxylase (*CYP2U1*), GPI‐anchor synthesis pathway (*PGAP1*), phospholipase A1 (*DDHD2*), arylsulfatase family member I (*ARSI*), phospholipase A1 (*DDHD1*), fatty acid‐2 hydroxylase (*FA2H*), neuropathy target esterase (*PNPLA6*), nonlysosomal glucosidase 2 (*GBA2*), GM2 synthase deficiency (*B46ALNT1*), serine active site containing 1 (*SERAC1*),^21^ ethanolaminephosphotransferase 1 (*EPT1*)^30^
NBIA	Calcium‐independent phospholipase A2γ (*PLA2G6*), pantothenate kinase 2 (*PANK2*), fatty acid‐2 hydroxylase (*FA2H*), CoA synthase deficiency (*COASY*), phospholipase A1 (*DDHD1*)^31^
Ataxia	Neuropathy target esterase (*PNPLA6*), coenzyme Q10 deficiency (*COQ8‐ADCK3*)
Movement disorders	Serine active site containing 1 (*SERAC1*), coenzyme Q deficiency (*COQ1‐PDSS2*), lactosylceramide α‐2,3 sialyltransferase (GM3 synthase; *ST3GAL5)*, calcium‐independent phospholipase A2γ (*PLA2G6*)
Complex or syndromic developmental delay/intellectual disability	Defects in the glycosylphosphatidylinositol–anchor biosynthesis pathway (*PGAP2, PGAP3, PIGA, PIGN, PIGL, PIGO, PIGT, PIGV, PIGW, PIGY*)^32^, ^33^
Epilepsy	Lactosylceramide α‐2,3 sialyltransferase (GM3 synthase; *ST3GAL5*), defects in the glycosylphosphatidylinositol–anchor biosynthesis pathway (*PIGM, PIGN, PIGA*)

Adapted and updated from Garcia‐Cazorla et al.^29^

NBIA = neurodegeneration with brain iron accumulation.

Currently, no effective treatment for MEGDHEL syndrome is available. Disease management options are directed toward proper supportive care.

## Author Contributions

Study concept and design: S.B.W., R.A.W., A.P.d.B. Data acquisition and analysis: K.I.‐P., S.K.U., B.A., M.A., M.A.A.‐O., H.I.A.‐Z., S.B., I.B., D.B., A.B., J.C., W.K.C., R.C., N.D., P.F., M.T.G.S., S.G., T.B.H., P.M.v.H., O.H., F.H., P.I., K.R., R.K.‐N., Z.K., E.M.‐H., J.A.M., P.M., L.D.M., K.N., L.H.N., M.P., S.R., G.R., L.R., B.R., F.R., R.S., M.Sc., M.Se., S.S., W.S., C.S., M.S., R.W.T., J.T., K.T., O.U., E.W., Y.W., T.W., H.P., E.M., E.P. Drafting manuscript and figures: R.R.M., S.B.W., R.A.W., A.P.d.B.

## Potential Conflicts of Interest

Nothing to report.

## Supporting information

Additional supporting information can be found in the online version of this article.

supporting information

supporting information

supporting information

supporting information
