# Supplementary figures and images for: Progressive deafness–dystonia due to SERAC1 mutations: A study of 67 cases
Source: Ann Neurol. 2017 Dec 20;82(6):1004–15. doi: 10.1002/ana.25110 (PMC5847115; doi:10.1002/ana.25110)

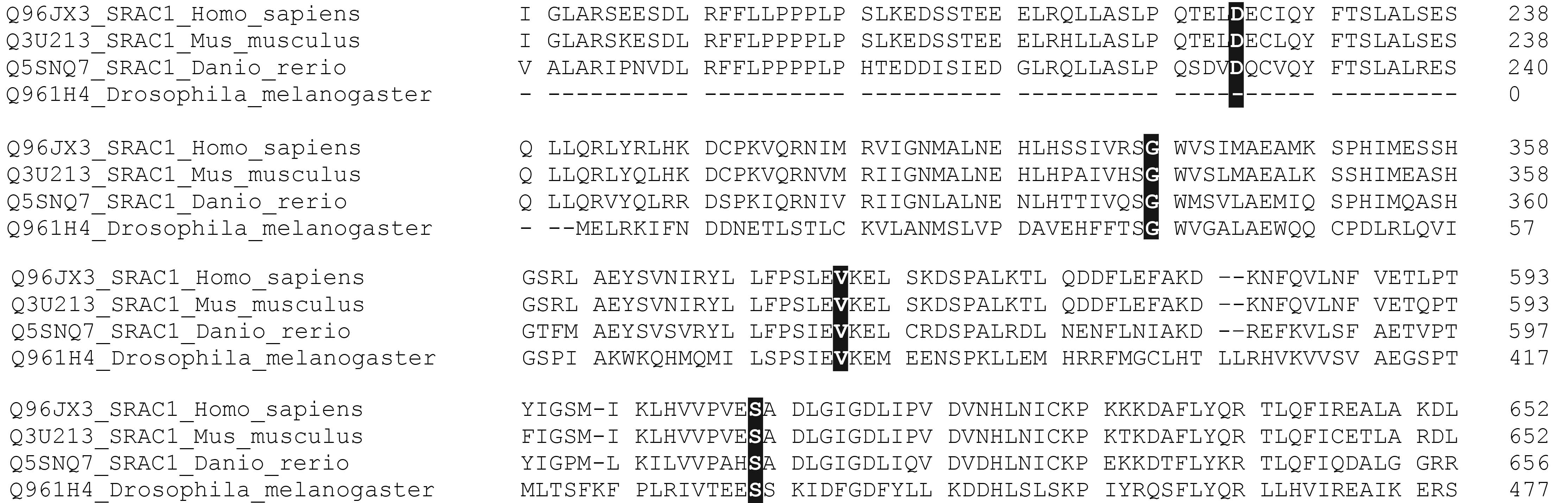

Supplement: Supplementary file 1 — supporting information [file ANA-82-1004-s001.jpg]
