# Supplementary material for: Progressive deafness–dystonia due to SERAC1 mutations: A study of 67 cases
Source: Ann Neurol. 2017 Dec 20;82(6):1004–15. doi: 10.1002/ana.25110 (PMC5847115; doi:10.1002/ana.25110)
Supplement: Supplementary file 2 — supporting information [file ANA-82-1004-s002.docx]

**Supplementary Table 1:** Variants found in *SERAC1*

| **Variant** | **Predicted effect on protein level** | **Type of variant** | **Predicted effect** | **Individual** | **Zygosity** | **Country of origin** | **Reference** |
| --- | --- | --- | --- | --- | --- | --- | --- |
| **deletion Ex4-8** | **p.Gly44Valfs*30** | **canonical splice site** | **LOF** | **18** | **heterozygous** | **Latvia** | **This paper** |
| **c.91A>T** | **p.Arg31*** | **nonsense** | **LOF** | **32** | **homozygous** | **Saudi Arabia** | **This paper** |
| **c.91+6T>C** | **p.?** | **splice site** | **LOF** | **(60-64)*** | **homozygous** | **Iraq** | ^21^ |
| **c.92delG** | **p.Arg31Lysfs*3** | **frameshift** | **LOF** | **43,44** | **homozygous** | **Saudi Arabia** | **This paper** |
| c.202C>T | p.Arg68* | nonsense | LOF | 16,47,50 | homozygous | Croatia, Spain, French African country | ^12^ |
| c.310A>T | p.Lys104* | nonsense | LOF | 49 | heterozygous | Portugal | ^20^ |
| c.438delC | p.Thr147Argfs*22 | frameshift | LOF | 58 | heterozygous | Saudi Arabia | ^14^ |
| c.442C>T | p.Arg148* | nonsense | LOF | (3,6,7)*, 21,33,34,38,58 | 3, 6, 7, 33, 34, 38 homozygous, 21, 58 heterozygous | Turkey, China, Saudi Arabia | ^2^ |
| c.456_466dup | p.Ser156Cysfs*17 | frameshift | LOF | 14 | heterozygous | Sweden | ^2^ |
| **c.547C>T** | **p.Arg183*** | **nonsense** | **LOF** | **20** | **heterozygous** | **Italy** | **This paper** |
| **c.576dup** | **p.Leu193Serfs*16** | **frameshift** | **LOF** | **26** | **homozygous** | **Malaysia** | **This paper** |
| c.576delT | p.Leu193Serfs*9 | frameshift | LOF | 15 | heterozygous | Sweden | ^2^ |
| c.609+5_609+8del | p.? | splice site | LOF | 49 | heterozygous | Portugal | ^20^ |
| c.671A>G | p.Asp224Gly | missense | ? | 66 | homozygous | Spain | ^15^ |
| **c.763_770dup** | **p.Pro258Metfs*22** | **frameshift** | **LOF** | **28** | **homozygous** | **South Africa** | **This paper** |
| c.800delC | p.Pro267Leufs*10 | frameshift | LOF | (51,52)* | homozygous | Turkey | ^13^ |
| **c.916C>T** | **p.Arg306*** | **nonsense** | **LOF** | **29, 59** | **heterozygous** | **German/Polish/Curacao, Australia** | **This paper** |
| **c.942delA** | **p.Gln315Argfs*4** | **frameshift** | **LOF** | **45** | **heterozygous** | **Belgium** | **This paper** |
| **c.1015G>C** | **p.Gly339Arg** | **missense** | **?** | **(53,54)*** | **homozygous** | **Turkey** | **This paper** |
| **c.1102C>T** | **p.Arg368*** | **nonsense** | **LOF** | **65** | **heterozygous** | **Germany** | **This paper** |
| **c.1112_1113del** | **p.Val371Alafs*22** | **frameshift** | **LOF** | **36** | **homozygous** | **Turkey** | **This paper** |
| c.1167_1170delTCAG | p.Gln390Profs*29 | frameshift/canonical splice site | LOF | 1 | homozygous | Turkey | ^2^ |
| c.1202G>A | p.Gly401Asp | missense | ILF | 13 | heterozygous | Sweden | ^2^ |
| c.1211G>A | p.Gly404Glu | missense | ILF | 8 | homozygous | Afghanistan | ^2^ |
| **c.1228T>C** | **p.Trp410Arg** | **missense** | **ILF** | **22,31** | **homozygous** | **Somalia** | **This paper** |
| c.1309_1313dup | p.Trp438* | nonsense | LOF | 10 | heterozygous | Poland | ^2^ |
| **c.1339C>T** | **p.Arg447*** | **nonsense** | **LOF** | **19** | **homozygous** | **Turkey** | **This paper** |
| c.1403+1G>C | p.? | canonical splice site | LOF | 5,12,42,46,55 | homozygous | Pakistan, India, Bangladesh, Turkey | ^2^ |
| **c.1403+4A>G** | **p.?** | **splice site** | **LOF** | **48** | **heterozygous** | **Rumania** | **This paper** |
| c.1435_1437delCTT | p.Leu479del | in frame deletion of single aa | ILF | 4 | homozygous | Turkey | ^2^ |
| c.1493G>C | p.Ser498Thr | missense | ILF | 14, 15, 27, 59 | 27 homozygous, 14,15,59 heterozygous | Sweden, Australia | ^2^ |
| c.1598_1599ins17 | p.Gly536Ilefs*56 | frameshift | LOF | 9 | homozygous | Turkey | ^2^ |
| c.1628_1629dupCT | p.Val544Leufs*43 | frameshift | LOF | 2 | homozygous | The Netherlands | ^2^ |
| **c.1642dup** | **p.Tyr548Leufs*20** | **frameshift** | **LOF** | **24** | **homozygous** | **Poland** | **This paper** |
| **c.1644T>G** | **p.Tyr548*** | **nonsense** | **LOF** | **25, 39** | **homozygous** | **Pakistan** | **This paper** |
| **c.1646_1647insAGAT** | **p.Leu550Aspfs*19** | **frameshift** | **LOF** | **40, 41, 45** | **40,41 homozygous¸ 45 heterozygous** | **Finland, Belgium** | **This paper** |
| **c.1667T>A** | p.Val556Asp | **missense** | **?** | **67** | **Homozygous** | **Turkey** | **This paper** |
| c.1822_1828+10delinsACCAACAGG | p.? | canonical splice site | Truncation of last 45 aa residues | 10, 11, 13, 17, 18, 20, 23, 29, 35, 37, 48, 56, 57, 65 | 17, 23, 35, 37, 56, 57 homozygous, 10, 11, 13, 18, 20, 29, 48, 65 heterozygous | Turkey, Latvia, Italy, Ukraine, Poland, Sweden, Rumania, Germany/Poland/Curacao, Germany | ^2^ |
| **c.1822T>A** | **p.Ser608Thr** | **missense** | **?** | **30** | **Homozygous** | **Turkey** | **This paper** |
| c.1924C>T | p.Gln642* | nonsense | Truncation of last 13 aa residues | 11 | Heterozygous | Poland | ^2^ |
| **c.1963dup** | **p.*655Leuext*32** | **extension** | **Extension of 32 aa residues** | **21** | **Heterozygous** | **Malaysian** | **This paper** |

Overview of all genetic variants found in the described individuals, references as in main text. The variants were found in either homozygous or compound heterozygous state. Previously unreported variants are shown in bold. * = individuals from one family, aa = amino acid, ILF=Impaired lipase function , LOF = loss of function, ? = unknown.
