# Supplementary material for: Progressive deafness–dystonia due to SERAC1 mutations: A study of 67 cases
Source: Ann Neurol. 2017 Dec 20;82(6):1004–15. doi: 10.1002/ana.25110 (PMC5847115; doi:10.1002/ana.25110)
Supplement: Supplementary file 3 — supporting information [file ANA-82-1004-s003.docx]

**Supplementary Table 2** Calculation of global incidence of MEGDEL syndrome

|  |  | Formula |  |
| --- | --- | --- | --- |
| *SERAC1* LoFs^*^ in the ExAC population (exac.broadinstitute.org^**^) | **(A)** |  | 41 |
| Proportion causal *SERAC1* missense:LoF variants (this paper) | **(B)** |  | 0.27 |
| Estimated amount of heterozygous causal alleles (missense + LoF) in ExAC | **(C)** | A+(A*B) | 52 |
| Estimated prevalence assuming that homozygous causal alleles do not result in early death | **(D)** | 1:1/(C/120000)^2^ | 1:5.34*10^6^ |
| Estimated incidence^#^ | **(E)** | (7.6*10^9^)/D*0.0187 | **27** |

^*^LoF: non-dubious stop, splice site, and frameshift variants in the canonical *SERAC1* transcript. All variants were heterozygous, ^**^approximately 120,000 *SERAC1* alleles were tested, ^#^ world population: 7.6 billion; births per person per year: 0.0187.
