# Supplementary material for: Progressive deafness–dystonia due to SERAC1 mutations: A study of 67 cases
Source: Ann Neurol. 2017 Dec 20;82(6):1004–15. doi: 10.1002/ana.25110 (PMC5847115; doi:10.1002/ana.25110)
Supplement: Supplementary file 4 — supporting information [file ANA-82-1004-s004.docx]

**Supplementary Table 3** Summary of individuals reported in the literature

|  | FamilyA/V-2/M^3^ | FamilyA/V-3/M^3^ | FamilyB/V-3/M^3^ | FamilyB/V-5/M^3^ | Individual^26^ | Index^18^ | Brother^18^ |
| --- | --- | --- | --- | --- | --- | --- | --- |
| Ethnicity | Arab Muslim | Arab Muslim | Druze | Druze | Palestine | Pakistan | Pakistan |
| Age of onset, course | 24-48h: hypotonia, NLF, ; 6 m failure to thrive, 13 m: deafness, seizures, spasticity, dystonia, regression | | | | Neonatal adaptation problems, H | NLF | NLF |
| *SERAC1* variant (homozygous) | c.698-9TG>AGTGATA | | c.128+4A>G | | c.1018delT | c.1403+1G>C | |
| Current age (deceased) | 9.5y | 4.5y | (5y) | (3.5y) | (2y). pneumonia | (12d) | 2y |
| Neonatal presentation | H, NLF | H, NLF | H, NLF | H, NLF | H | LF | LF |
| Muscular hypotonia, spasticity, dystonia | +, +, + | +, +, + | +, +, + | +, +, + | +, -, - | n/a | n/a |
| Oropharyngeal dyskinesia | n/a | n/a | n/a | n/a | n/a | n/a | n/a |
| Ability to walk | n/a | n/a | n/a | n/a | - | n/a | n/a |
| Intellectual disability | n/a | n/a | n/a | n/a | Severe | n/a | n/a |
| Epilepsy | + | + | + | + | - | - | n/a |
| MRI findings | LL | LL | LL | LL | LL (put. eye+) | n/a | n/a |
| Hearing loss | + | + | + | + | +, hearing aids | n/a | n/a |
| Optic atrophy | n/a | n/a | n/a | n/a | - | n/a | n/a |
| 3-MGA (mmol/mol creatinine) | 34-203 | 34-203 | 34-203 | 34-203 | Moderate | + | n/a |
| Lactic acidosis | + | + | + | + | + | + | n/a |
| Muscle biopsy | n/a | n/a | n/a | * | n/a | n/a | n/a |
| Liver biopsy | ** | | | | n/a | n/a | n/a |
| Filipin staining | 2/2 individuals + | | | | n/a | n/a | n/a |

3-MGA= 3-methylglutaconic aciduria, H= hypoglycaemia, LL=Leigh-like, n/a = not available, NLF = neonatal liver failure. *Histology, immunohistochemistry, electron microscopy unremarkable, **3/3 micro-macrosteatosis, electron microscopy 1/1 ultrastructural changes of the mitochondria, 1/1 mitochondrial DNA depletion. References as in main text.
